# Circulating cytotoxic immune cell composition, activation status and toxins expression associate with white matter microstructure in bipolar disorder

**DOI:** 10.1038/s41598-023-49146-6

**Published:** 2023-12-14

**Authors:** Veronica Aggio, Lorena Fabbella, Sara Poletti, Cristina Lorenzi, Annamaria Finardi, Cristina Colombo, Raffaella Zanardi, Roberto Furlan, Francesco Benedetti

**Affiliations:** 1 Psychiatry and Clinical Psychobiology Unit, Division of Neurosciences, IRCCS San Raffaele Scientific Institute, San Raffaele Turro, Via Stamira d’Ancona 20, 20127 Milano, Italy; 2 Vita-Salute San Raffaele University, Milan, Italy; 3 Clinical Neuroimmunology Unit, Division of Neuroscience, Institute of Experimental Neurology, IRCCS San Raffaele Scientific Institute, Milan, Italy; 4 Mood Disorders Unit, IRCCS Scientific Institute Ospedale San Raffaele, Milano, Italy

**Keywords:** Immunology, Neuroscience, Biomarkers

## Abstract

Patients with bipolar disorder (BD) show higher immuno-inflammatory setpoints, with in vivo alterations in white matter (WM) microstructure and *post-mortem* infiltration of T cells in the brain. Cytotoxic CD8^+^ T cells can enter and damage the brain in inflammatory disorders, but little is known in BD. Our study aimed to investigate the relationship between cytotoxic T cells and WM alterations in BD. In a sample of 83 inpatients with BD in an active phase of illness (68 depressive, 15 manic), we performed flow cytometry immunophenotyping to investigate frequencies, activation status, and expression of cytotoxic markers in CD8^+^ and tested for their association with diffusion tensor imaging (DTI) measures of WM microstructure. Frequencies of naïve and activated CD8^+^ cell populations expressing Perforin, or both Perforin and Granzyme, negatively associated with WM microstructure. CD8^+^ Naïve cells negative for Granzyme and Perforin positively associates with indexes of WM integrity, while the frequency of CD8^+^ memory cells negatively associates with index of WM microstructure, irrespective of toxins expression. The resulting associations involve measures representative of orientational coherence and myelination of the fibers (FA and RD), suggesting disrupted oligodendrocyte-mediated myelination. These findings seems to support the hypothesis that immunosenescence (less naïve, more memory T cells) can detrimentally influence WM microstructure in BD and that peripheral CD8^+^ T cells may participate in inducing an immune-related WM damage in BD mediated by killer proteins.

## Introduction

Bipolar disorder (BD) has been consistently associated with alterations in the immune system^1^. Evidence suggests a condition of systemic low-grade inflammation due to decreased adaptive and increased innate immunity, with higher levels of circulating cytokines, higher macrophage/monocyte inflammatory activation patterns, and higher neutrophils to lymphocyte counts; and with a dynamic pattern of premature immunosenescence and partial T cell defect starting early in adolescence, involving a reduction of naïve T cells and an expansion of memory and senescent T cells^2–6^. Quantitative analysis of circulating inflammatory markers, such as cytokines and chemokines, suggested persistent low-grade inflammation^7,8^. Measured in peripheral blood, these markers of altered immuno-inflammatory setpoints parallel activation of microglia and disruption of white matter (WM) integrity in the brain^9,10^. Moreover, *post-mortem* studies documented abnormal T and B cell infiltration of brain tissue in subgroups of patients with mood disorders, but their possible pathogenetic role is largely unknown^11^.

Altered WM microstructure in circuitries critical for emotional and cognitive processing is a core biological correlate of BD^12^. In vivo diffusion tensor imaging (DTI) consistently showed higher mean diffusivity of water (MD), with higher diffusivity perpendicular to the main axis of fibers (radial diffusivity, RD, mainly associated with myelination), and lower diffusivity along the main axis (axial diffusivity, AD, mainly associated with the axonal microstructure), resulting in lower preferential diffusivity along WM tracts (fractional anisotropy, FA). These measures reflect myelination, orientational coherence, and microtubular axonal structure of fibers^13–15^. In BD, they associate with genetic risk^16,17^, with environmental stressors increasing the disorder risk^18^, with markers of and with gene variants affecting the course and outcome of the illness^19–21^; and underpin highly relevant clinical phenotypes such as impulsivity and suicide^22^, cognitive performance^23,24^, and response to treatment^25^.

A growing literature suggests that the immune system plays a core role in maintaining brain homeostasis, with both adaptive and innate immune support, ensured by cell trafficking across the blood–brain barrier, being essential for brain maintenance and repair in healthy conditions and disrupted in brain disorders including BD^26,27^. It can be hypothesized that the circulating immune cell composition and activation status, reflecting individual variation and altered immunophenotyping associated with BD, could also be associated with altered brain phenotypes, such as disrupted WM microstructure. Studies in the field are in its infancy, but previous findings by our group confirm that the balance between cell subsets might play a role in the maintenance of the structural brain integrity in BD: circulating Th17 cells correlated with higher FA, while regulatory FOXP3^+^ cells correlated with higher RD and MD, and with lower fMRI neural responses in the right dorsolateral prefrontal cortex^2^, a phenotype associated with bipolar depression^28^; higher circulating cytokine-producing NK cells (TNFα, INFγ, and GMCSF) were fostered by ongoing lithium treatment and directly correlated with better FA, and inversely with RD and MD, also partially mediating the known benefits from lithium on WM^29,30^; levels of the cytokines TNF-α, IL-8, IFN-γ, and IL-10 associated negatively with AD and positively with RD, suggesting that markers of peripheral immune-inflammatory activation negatively affect the integrity of myelin sheaths^31^.

However, the majority of circulating leukocyte populations associated with BD has not yet been studied in this perspective. Among them, cytotoxic T lymphocytes (CTLs) (CD3^+^CD8^+^) play a crucial role in the adaptive immune response, and stimulation by antigens throughout life markedly favors their switching towards senescent phenotypes thus contributing to low-grade inflammation: it is suggested that a better characterization of their phenotype and functions could be pivotal to prevent immune-related medical conditions^32^. In BD, the few available studies reported either no difference in the CD8^+^ compartment in respect to healthy controls^33^; or a general decrement in the cytotoxic T cells compartment^34,35^, a decrease in replicating/proliferating (CD71^+^) cells^36^, and an increase in proportions of late-differentiated cells (CD28^−^CD27^−^) associated both, with age, and with recurrence of illness episodes^37^. A single study showed a general depletion of the CD8^+^ compartment, and positively associated activated CD8^+^IFNγ^+^ and activated CD8^+^CD28^−^CD45RA^+^ cells with increased FA and decreased RD, in corona radiata and corpus callosum, during manic illness episodes, and not during depression or euthymia^38^.

Cytotoxic CD8^+^ T cells can enter the brain, and are considered important effector cells contributing to neuronal damage in inflammatory and degenerative brain disorders^39^. In BD, they appear to be more activated and senescent, their proportion also influencing WM integrity in some illness phases, but data on their activation status and expression of cytotoxic markers are lacking.

The present study aims to investigate a possible specific involvement of the lymphocytes cytotoxic compartment associated to previously reported WM alterations. To do that, we aimed at associating, with a comprehensive and systematic approach, TBSS measures of WM integrity with CD8^+^, NK, and Tγδ cells immunophenotyping data of 83 patients with an active illness episode in course of BD.

## Methods

### Participants

All participants to this study were volunteers: each subject was provided with a complete description of the study and gave written informed consent for participation. All the experimental protocols and procedures contributing to this work comply with the ethical standards, relevant guidelines and regulations of the relevant national and institutional committees on human experimentation and with the Helsinki Declaration of 1975, as revised in 2008. The study was approved by the local ethical committee of San Raffaele Hospital.

Out of 102 screened inpatients with BD, consecutively admitted to the Center for Mood Disorders of the San Raffaele Hospital, 83 biologically unrelated inpatients (F 55; M 28) with a diagnosis of Bipolar Disorder Type I (DSM-5 criteria) were included in the study: 68 patients had a depressive and 15 a manic episode. All the patients enrolled underwent a psychiatric interview and a physical examination performed by a resident psychiatrist of the ward. The tests performed for the diagnostic evaluation were the nationally validated versions of the Hamilton depression rating scale (HDRS)^40^, young mania rating scale (YMRS)^41^, and beck depression inventory (BDI)^42^. Inclusion criteria were: to agree to participate; age between 18 and 65 years; signed informed consent. Exclusion criteria were: other psychiatric diagnoses, current pregnancy, history of epilepsy, major neurological or physical illnesses, previous cranial and/or cerebral injuries, autoimmune disorders, intellectual disability, current inflammatory conditions (e.g., infections, viruses) or anti-inflammatory drugs use (steroidal and non-steroidal), and history of alcohol and/or substance abuse in the lasts 6 months. Nicotine was not considered an abuse substance within the exclusion criteria.

### Laboratory methods

The immunophenotype of T cytotoxic lymphocyte subpopulations was obtained by flow cytometry on each participant's isolated peripheral blood mononuclear cells (PMBCs). The blood sampling was performed in the morning (8.00–9.00 AM). PBMCs were immediately (within 1 h from the blood collection) isolated by density gradient centrifugation with Ficoll-Paque PLUS (GE Healthcare, Uppsala, Sweden), and cryo-preserved in a freezing medium with 90% Fetal Bovine Serum (FBS) (Thermo Fisher) and 10% dimethyl sulfoxide (DMSO) in liquid nitrogen at − 192° until flow cytometry. Both surface and intracellular stainings were performed to characterize the cytotoxic activity of T-lymphocytes (CD8 +), T ϒδ lymphocytes and NK cells. After morphological characterization, we used the following gating strategy: the expression of CD3 and CD56 markers were used to identify and gate both T-lymphocytes (CD3^+^CD56^−^) and NK cells (CD3^−^CD56^+^). On CD3^+^CD56^−^ gated cells, the relative expression of CD4 and CD8 was used to identify CD8^+^ lymphocytes. Moreover, the relative expression of Vd1 and Vd2 was used to identify T ϒδ lymphocytes (CD3^+^Vd1Vd2^+^). On CD8^+^ T cells, further characterization of primed (memory) cells was performed using CD27 and CD45RA markers: naϊve cells express both cell surface glycoproteins (CD45RA^+^CD27^+^), central memory cells express CD27 but not CD45RA (CD45RA^−^CD27^+^), effector memory (EM) cells are double negative (CD45RA^−^CD27^−^), and terminally differentiated effector memory cells re-express CD45RA (TEMRA) cells (CD45RA^+^CD27^−^). Finally, granzyme and perforin relative expression was analyzed on NK cells, differentially primed CD8^+^ T-cells, and T ϒδ lymphocytes to evaluate the activation of cytotoxic mechanisms. Blood was sampled in the morning on the same day of the MRI study. Cell frequencies were compared among the two groups of patients with a manic or a depressive episode by analyzing the homogeneity of slopes in the context of a generalized linear model (GLZM), with an identity link function^43^. Parameter estimates were obtained with iterative re-weighted least squares maximum likelihood procedures. The significance of the effects was calculated with the likelihood ratio (LR) statistic, which provides the most asymptotically efficient test known^44,45^.

### Image acquisition and analysis

DWI was executed using SE Eco-planar imaging (EPI) and the following parameters for the first scanner TR/TE = 8753.89/58 ms, FoV (mm) 231.43 (ap), 126.50 (fh), 240.00 (rl); acquisition matrix 2.14 × 2.71 × 2.31; 55 contiguous, 2.3 mm thick axial slices reconstructed with in-plane pixel size 1.88 × 1.87 mm; SENSE acceleration factor = 2; 1 b0 and 35 non-collinear directions of the diffusion gradients; b value = 900 s/mm^2^; and the following for the second Philips scanner TR/TE = 5900/78 ms, FoV (mm) 240 (ap), 129 (fh), 232 (rl); acquisition matrix 2.14 × 2.73 × 2.30; 56 contiguous, 2.3 mm thick axial slices reconstructed with in-plane pixel size 1.88 × 1.88 × 2.30 mm; SENSE acceleration factor = 2; 1 b0 and 40 non-collinear directions of the diffusion gradients; b value = 1000 s/mm^2^. Fat saturation was performed to avoid chemical shift artifacts.

DTI analysis and tensor calculations were carried out using the "Oxford Center for Functional Magnetic Resonance Imaging of the Brain Software Library" (FSL 6.0; www.fmrib.ox.ac.uk/fsl/index.html)^46^.

Each DWI volume was affine registered to the T2-weighted b = 0 volume using FLIRT (FMRIB's Linear Image Registration Tool). Then, correction for motion between scans and residual eddy-current distortions present in the diffusion-weighted images was performed. After removal of nonbrain tissue, least-square fits were performed to estimate the FA, eigenvector, and eigenvalue maps. MD was defined as the mean of all three eigenvalues (λ_1_^+^ λ_2_^+^ λ_3_)/3, AD as the principal diffusion eigenvalue (λ_1_), and RD as the mean of the second and third eigenvalues (λ_2_^+^ λ_3_)/2. Next, all individuals' volumes were skeletonized and transformed into a common space as used in Tract-Based Spatial Statistics^47^. Briefly, all volumes were nonlinearly warped to the FMRIB58_FA template supplied with FSL (http://www.fmrib.ox.ac.uk/fsl/tbss/FMRIB58_FA.html) and normalized to the montreal neurological institute (MNI) space, by use of local deformation procedures performed by FMRIB's non-linear image registration tool (FNIRT). Next, a mean FA volume of all subjects was generated and thinned to create a mean FA skeleton representing the centers of all common tracts. We thresholded and binarized the mean skeleton at FA > 0.20 to reduce the likelihood of partial voluming in the borders between tissue classes. Individual FA values were warped onto this mean skeleton mask. The resulting tract invariant skeletons for each participant were fed into voxel-wise permutation-based cross-subject statistics. Similar warping and analyses were used on MD, AD, and RD data.

We accounted for the effects of nuisance covariates that could influence WM microstructure: age^48^, sex^49^, lithium treatment^30^, phase of illness, and MR scanner. MRI scan variable has been created as a dichotomous variable, which can assume one of two possible values in accordance with the MRI scan used for each subject. This has been done in order to control for the potential confounding effect of using two different scanners in the analyses. Voxel-wise DTI analyses were performed using nonparametric permutation-based testing as implemented in Randomise in FSL. We tested for linear effects of the concentration of each cellular subpopulation on FA, MD, AD, and RD across the WM skeleton with general linear models (GLM). Threshold-free cluster enhancement (TFCE) was used to avoid defining arbitrary cluster-forming thresholds and smoothing levels. The data were tested against an empirical null distribution generated by 5000 permutations for each contrast, thus providing statistical maps fully corrected for multiple comparisons across space. Corrected *p* < 0.05 was considered significant.

To better understand a possible effect of the illness phase (manic or depressed), we also performed the same DTI analyses in the subsample of sixty-eight depressed patients, excluding those with a manic episode, and maintaining age, sex, lithium treatment, and MR scanner as nuisance covariates.

Finally, for each significant analysis, we extracted the mean values of the DTI measures for each subject in the significant WM tracts were the signal peak was observed, selected through the JHU-ICBM-DTI label atlas. Then, we performed a regression analysis within the GLZM framework to evaluate the strength of the association of each significant imaging result (Supplementary information).

## Results

The clinical and demographic characteristics of the sample are reported in Table 1. The mean and standard deviation of all the observed relative frequencies of the different cellular subpopulations obtained through FACS immunophenotyping are reported in Table 2. Patients with a depressive episode were younger, and with a younger age at onset of illness than patients with a manic episode. Cell frequencies were not significantly different among groups, correcting for age, sex, and age at onset of illness. The cell frequencies did not correlate with illness duration and number of episodes. In the whole sample with both depressed and manic patients, circulating cytotoxic T-cell frequencies and activation status were significantly associated with WM microstructure (Table 3). In general, compared to the whole panel analyzed, we observed that only the frequencies of Naïve, EM, and CM cells expressing Perforin and Granzyme were associated with measures of reduced WM integrity (reduced FA and/or increased RD and MD), while frequencies of Naïve and CM cells negative for Perforin and Granzyme associated with measures of better WM integrity (increased FA and reduced RD). More in detail, the cells that were associated with measures of WM integrity were CD8^+^ Naïve Perf^−^Grz^−^ and CD3^+^CD8^+^EM Perf^−^Grz^+^ that positively associated with FA and negatively with RD; CD3^+^CD8^+^CM Perf^−^Grz^−^ that negatively associated with RD and MD; CD3^+^CD8^+^TEMRA Perf^−^Grz^+^ that positively associated with FA measures. The cell subpopulations that were related to measures of reduced WM microstructural integrity were: CD3^+^CD8^+^Naïve Perf^+^Grz^−^ and CD3^+^CD8^+^CM Perf^−^Grz^+^, which negatively associated with FA and positively associated with both RD and MD; CD3^+^CD8^+^Naïve Perf^+^Grz^+^, CD3^+^CD8^+^EM Perf^+^Grz^−^, CD3^+^CD8^+^CM Perf^+^Grz^−^, and CD3^+^CD8^+^TEMRA Perf^+^Grz^−^ which negatively associated with FA and positively associated with RD; finally, CD3^+^CD8^+^CM, CD3^+^CD8^+^EM Perf^+^Grz^+^ and CD3^+^ CD8^+^CM Perf^+^Grz^+^ which resulted negatively associated with FA. No significant effect was observed on AD measures. NK and Tγδ cells were not related to structural brain measures.

**Table 1:** Clinical and demographic characteristics of participants, and level of significance (t-test) of the differences observed according to the polarity of the acute illness episode.

	Depressive episode (n = 68)	Manic episode (n = 15)	T-test /Pearson's Chi-square		All patients
N = 83	Mean ± SD	Mean ± SD	t-value	*P*	Mean ± SD
Age	46.45 ± 11.43	53.13 ± 11.48	2.05	*0.044	47.66 ± 11.66
Sex	50 (F) and 18 (M)	5 (F) and 10 (M)	8.88	*0.029	55 (F) and 28 (M)
Age at illness onset	28.4 ± 10.05	38.4 ± 12.76	3.31	*0.001	30.2 ± 11.2
Education (year in school)	13.02 ± 3.95	12.2 ± 3.73	0.73	0.47	12.83 ± 3.93
Illness duration (in years)	18.05 ± 11.16	14.73 ± 11.6	1.04	0.302	17.46 ± 11.24
Duration of the current episode (in weeks)	30.63 ± 46.74	7.53 ± 6.66	1.9	0.06	26.46 ± 43.28
n° of depressive episodes	6.02 ± 5.27	4.29 ± 3.99	1.15	0.25	5.71 ± 5.09
n° of manic episodes	3.17 ± 3.62	4.07 ± 4.03	3.17	0.41	3.33 ± 3.68
Lithium treatment in the previous 6 months	36 (no) vs 31 (yes)	7 (no) vs 8 (yes)			44 (no) versus 39 (yes)
Total months under lithium treatment	45,7 ± 60.94	45.5 ± 66.08	0.011	0.99	45.67 ± 61.42
HDRS	19.58 ± 6.49	0.08 ± 0.29	10.34	*0.000	16.55 ± 9.29
YOUNG	0.00 ± 0.00	18.62 ± 11.88	11.58	*0.000	3.72 ± 9.09
BDI	14.21 ± 8.11	6.21 ± 7.79	3.23	*0.002	12.25 ± 8.7
Body mass index	26.82 ± 5.26	27.09 ± 3.87	0.18	0.85	26.87 ± 5.03

*HDRS* hamilton depression rating scale, YOUNG young mania rating scale, *BDI* beck depression inventory.

**Table 2:** Cytotoxic CD8^+^ cell frequencies observed in participants, and levels of significance of the observed differences (GLZM homogeneity of slopes, likelihood ratio χ^2^, corrected for age, sex, and age at onset of illness).

Cytotoxic cells	Depressive episode (n = 68)	Manic episode (n = 15)	LR χ^2^	*p*	Whole sample (n = 83)
CD3^+^	65.5 ± 10.2	71.9 ± 7.6	0.085	0.77	66.6 ± 10
CD3^+^CD4^+^	69.1 ± 8.7	68.2 ± 12.2	0.070	0.79	68.9 ± 9.3
CD3^+^CD8^+^	25.3 ± 8.4	24.8 ± 7.8	0.079	0.78	25.2 ± 8.3
CD3^+^CD8^+^NAIVE^+^	35.4 ± 19.2	32.3 ± 20.9	0.027	0.87	34.9 ± 19.5
CD3^+^CD8^+^NAIVE^+^Perf^−^Grz^+^	2.1 ± 2.6	4.1 ± 7.5	3.398	0.07	2.4 ± 3.9
CD3^+^CD8^+^NAIVE^+^Perf^+^Grz^+^	2.5 ± 4.5	1.9 ± 2.9	0.026	0.87	2.4 ± 4.2
CD3^+^CD8^+^NAIVE^+^Perf^+^Grz^−^	2.1 ± 3.5	2.3 ± 3.6	0.070	0.79	2.2 ± 3.6
CD3^+^CD8^+^NAIVE^+^Perf^−^Grz^−^	93.1 ± 7.3	91.6 ± 8.3	0.237	0.63	92.8 ± 7.5
CD3^+^CD8^+^EM^+^	16.6 ± 14.1	22.1 ± 16.5	2.398	0.12	17.6 ± 14.6
CD3^+^CD8^+^EM^+^Perf^−^Grz^+^	23.6 ± 18.6	32.1 ± 20.4	2.351	0.13	25.1 ± 19.1
CD3^+^CD8^+^EM^+^Perf^+^Grz^+^	12.6 ± 12.9	15.4 ± 18.7	0.054	0.82	13.1 ± 14
CD3^+^CD8^+^EM^+^Perf^+^Grz^−^	5.2 ± 6.9	4.2 ± 6.6	0.121	0.73	5 ± 6.8
CD3^+^CD8^+^EM^+^Perf^−^Grz^−^	58.6 ± 19.9	48.3 ± 21.2	2.127	0.14	56.8 ± 20.4
CD3^+^CD8^+^CM^+^	33.1 ± 16.9	28.7 ± 17.3	1.188	0.27	32.3 ± 17
CD3^+^CD8^+^CM^+^Perf^−^Grz^+^	5.6 ± 7.3	6.6 ± 7.6	0.000	0.98	5.8 ± 7.3
CD3^+^CD8^+^CM^+^Perf^+^Grz^+^	4.2 ± 5.1	4.2 ± 5.5	0.067	0.79	4.2 ± 5.2
CD3^+^CD8^+^CM^+^Perf^+^Grz^−^	6.9 ± 9	7.2 ± 10.4	0.200	0.65	6.9 ± 9.2
CD3^+^CD8^+^CM^+^Perf^−^Grz^−^	83.3 ± 13	81.9 ± 15.3	0.161	0.69	83.1 ± 13.4
CD3^+^CD8^+^TEMRA^+^	10.7 ± 9.2	11.8 ± 9.2	0.086	0.77	10.9 ± 9.1
CD3^+^CD8^+^TEMRA^+^Perf^−^Grz^+^	23.2 ± 22.9	28.5 ± 23.3	0.246	0.62	24.2 ± 22.9
CD3^+^CD8^+^TEMRA^+^Perf^+^Grz^+^	28.9 ± 28.3	27.8 ± 29.1	1.039	0.31	28.7 ± 28.3
CD3^+^CD8^+^TEMRA^+^Perf^+^Grz^−^	3.3 ± 5.6	2.4 ± 3.6	0.358	0.55	3.1 ± 5.3
CD3^+^CD8^+^TEMRA^+^Perf^−^Grz^−^	44.6 ± 31.2	41.3 ± 33.1	1.914	0.17	43.9 ± 31.3
CD3^+^Tγδ^+^	4.6 ± 6.9	2.3 ± 1.6	0.440	0.51	4.2 ± 6.4
CD3^+^Tγδ^+^Perf^−^Grz^+^	10.1 ± 10.2	14.2 ± 11.6	1.469	0.23	10.9 ± 10.5
CD3^+^Tγδ^+^Perf^+^Grz^+^	13.1 ± 14.6	12.4 ± 15.2	0.026	0.87	12.9 ± 14.6
CD3^+^Tγδ^+^Perf^+^Grz^−^	10.4 ± 14.7	5.9 ± 7.8	0.319	0.57	9.6 ± 13.8
CD3^+^Tγδ^+^Perf^−^Grz^−^	66.4 ± 22.7	67.5 ± 20.4	0.629	0.43	66.6 ± 22.2
CD3^+^NK^+^	7.7 ± 4.7	6.2 ± 2.6	0.008	0.93	7.5 ± 4.4
CD3^−^NK^+^Perf^−^Grz^+^	18.4 ± 19.8	25.3 ± 25.6	1.289	0.26	19.6 ± 20.9
CD3^−^NK^+^Perf^+^Grz^+^	45.9 ± 31.7	40.8 ± 31.8	0.084	0.77	45 ± 31.6
CD3^−^NK^+^Perf^+^Grz^−^	10.2 ± 14.5	7.6 ± 12.5	0.309	0.58	9.8 ± 14.1
CD3^−^NK^+^Perf^−^Grz^−^	25.4 ± 22.6	26.4 ± 22.6	1.302	0.25	25.6 ± 22.4

Cells percentage details in table was derived through a sequential identification with the FlowJo v 10.7.1 software of the clustering performed from the gating strategy on FACS Symphony A5 Cell Analyzer.

**Table 3:** A summary of the principal correlation found between T lymphocyte subpopulations and TBSS indexes of WM microstructure.

Cell subpopulation	FA correlation, signal peak, and GLZM results (Chi-square, *p*)	RD correlation, signal peak, and GLZM results (Chi-square, *p*)	MD correlation, signal peak, and GLZM results (Chi-square, *p*)
CD3^+^ CD8^+^ CM	↓, R Posterior Thalamic Radiation, (5.139, *p* < 0.05)		
CD3^+^ CD8^+^ CM Perf^−^Grz^+^	↓, L Posterior Limb of Internal Capsule, (7.488, *p* < 0.001)	↑, R Superior Longitudinal Fasciculus, (7.785, *p* < 0.005)	↑, (0.96), R Inferior Fronto-occipital Fasciculus (1.639, *p* = 0.200)
CD3^+^ CD8^+^ CM Perf^+^Grz-	↓, Genu of Corpus Callosum (5.843, *p* < 0.05)	↑, Genu of Corpus Callosum, (5.071, *p* < 0.05)	
CD3^+^ CD8^+^ CM Perf^+^Grz^+^	↓, L Anterior Corona Radiata, (3.926, *p* < 0.05)		
CD3^+^ CD8^+^ CM Perf^−^Grz^−^		↓, (0.99), Body of Corpus Callosum (3.518, *p* < 0.05)	↓, R Anterior Corona Radiata, (5.493, *p* < 0.05)
CD3^+^ CD8^+^ EM Perf^+^Grz^+^	↓, R Anterior Corona Radiata, (11.199, *p* < 0.001)		
CD3^+^ CD8^+^ EM Perf^+^Grz^−^	↓, Body of Corpus Callosum (14.547, *p* < 0.001)	↑, L Anterior Corona Radiata (10.634, *p* < 0.001)	
CD3^+^ CD8^+^ EM Perf^−^Grz^+^	↑, R Anterior Corona Radiata (4.900, *p* < 0.05)	↓, L Posterior Thalamic Radiation, (8.938, *p* < 0.005)	
CD3^+^ CD8^+^ Naïve Perf^+^Grz^+^	↓, R Cerebral Peduncle (3.869, *p* < 0.05)		
CD3^+^ CD8^+^ Naïve Perf^−^Grz^−^	↑, R Posterior Limb of Internal Capsule, (8.779, *p* < 0.005)	↓, R Anterior Limb of Interal Capsule, (4.173, *p* < 0.05)	
CD3^+^ CD8^+^ Naïve Perf^+^Grz^−^	↓, R Posterior Thalamic Radiation, (7.153, *p* < 0.01)	↑, Splenium of Corpus Callosum, (5.995, *p* < 0.05)	
CD3^+^ CD8^+^ TEMRA Perf^−^Grz^+^	↑, (0.97), L Anterior Corona Radiata (7.496, *p* < 0.01)		
CD3^+^ CD8^+^ TEMRA Perf^+^Grz^−^	↓, R Anterior Corona Radiata (7.190, *p* < 0.01)	↑, L Superior Corona Radiata (7.190, *p* < 0.01)	

For each TBSS index (Fractional Anisotropy, FA; Radial Diffusivity, RD; Mean Diffusivity, MD), the direction of the correlation (↑ positive correlation; ↓ negative correlation), the brain anatomical localization of the signal peak, and the strength of the association evaluated with GLZM statistical analyses performed on the extracted values of the WM tract of the signal peak.

**Figure 1:**
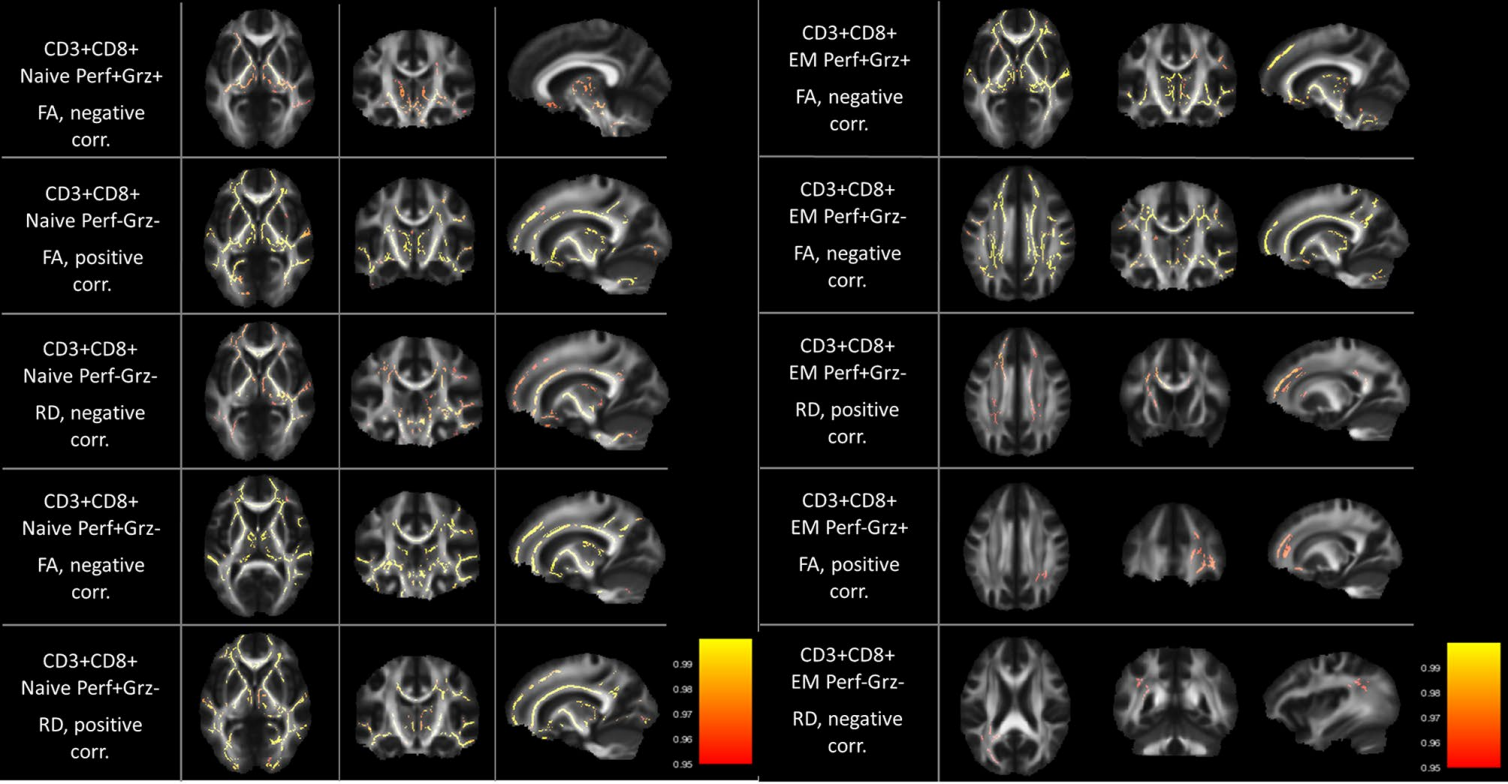
WM areas where CD3^+^CD8^+^EM and CD3^+^CD8^+^Naïve cells subpopulations percentage correlated with TBSS WM indexes. Voxels of significant negative correlation are mapped on the mean FA template of the studied sample. The colour-bar refers to 1 − *p* values for the observed differences. Numbers are z coordinates in the standard MNI space.

**Figure 2:**
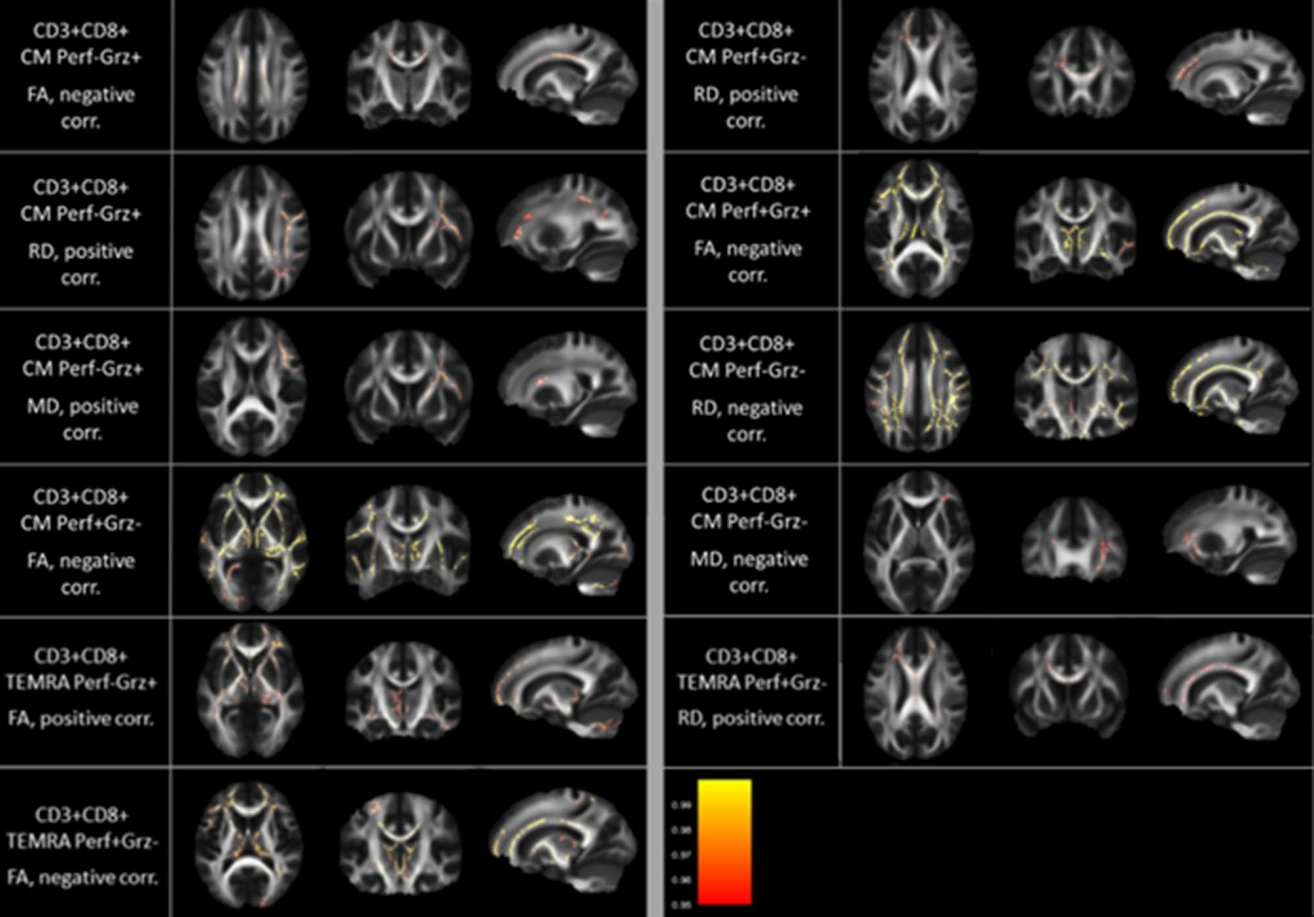
WM areas where CD3^+^CD8^+^CM cells and CD3^+^CD8^+^TEMRA cells subpopulations percentage correlated with TBSS WM indexes. Voxels of significant negative correlation are mapped on the mean FA template of the studied sample. The colour-bar refers to 1 − *p* values for the observed differences. Numbers are z coordinates in the standard MNI space.

Effects were spread in the WM skeleton for Naïve, EM (Fig. 1), CM, and TEMRA (Fig. 2) cells, with signal peaks observed in Thalamic Radiation, Corona Radiata, Corpus Callosum, Superior and Inferior Fronto-Occipital Fasciculus, Forceps Minor, Internal Capsule (see detailed summary of the results in Tables 2 and 3).

Analyzing depressed patients separately did not change the observed results.

## Discussion

The main finding of the present study is that differential expression of Perforin and Granzyme by CTLs is associated with in vivo measures of WM microstructure in patients with an active illness episode (depressive or manic) in the course of BD. While the frequency of naïve cells negative for both Perforin and Granzyme was associated with better WM integrity, frequencies of naïve and activated CD8^+^ cell populations positive for Perforin or both Perforin and Granzyme were negatively associated with WM microstructure. Effects were observed for measures associated with orientational coherence and myelination of the fibers (FA and RD) and not for measures associated with integrity and microtubular structure of the axons (AD), thus suggesting oligodendrocyte-mediated myelination as the main involved mechanism. The same results were confirmed in the depressive state subgroup. For this reason, it seems plausible to exclude, at this level, a possible effect of the episode (depressive or manic) in the relation between TBSS indexes and immune cell type. Moreover, this seems in line with the statistics analysis confirming no cell frequency differences among groups. For this reason, we will discuss the results reasoning on the whole group.

**Figure 3:**
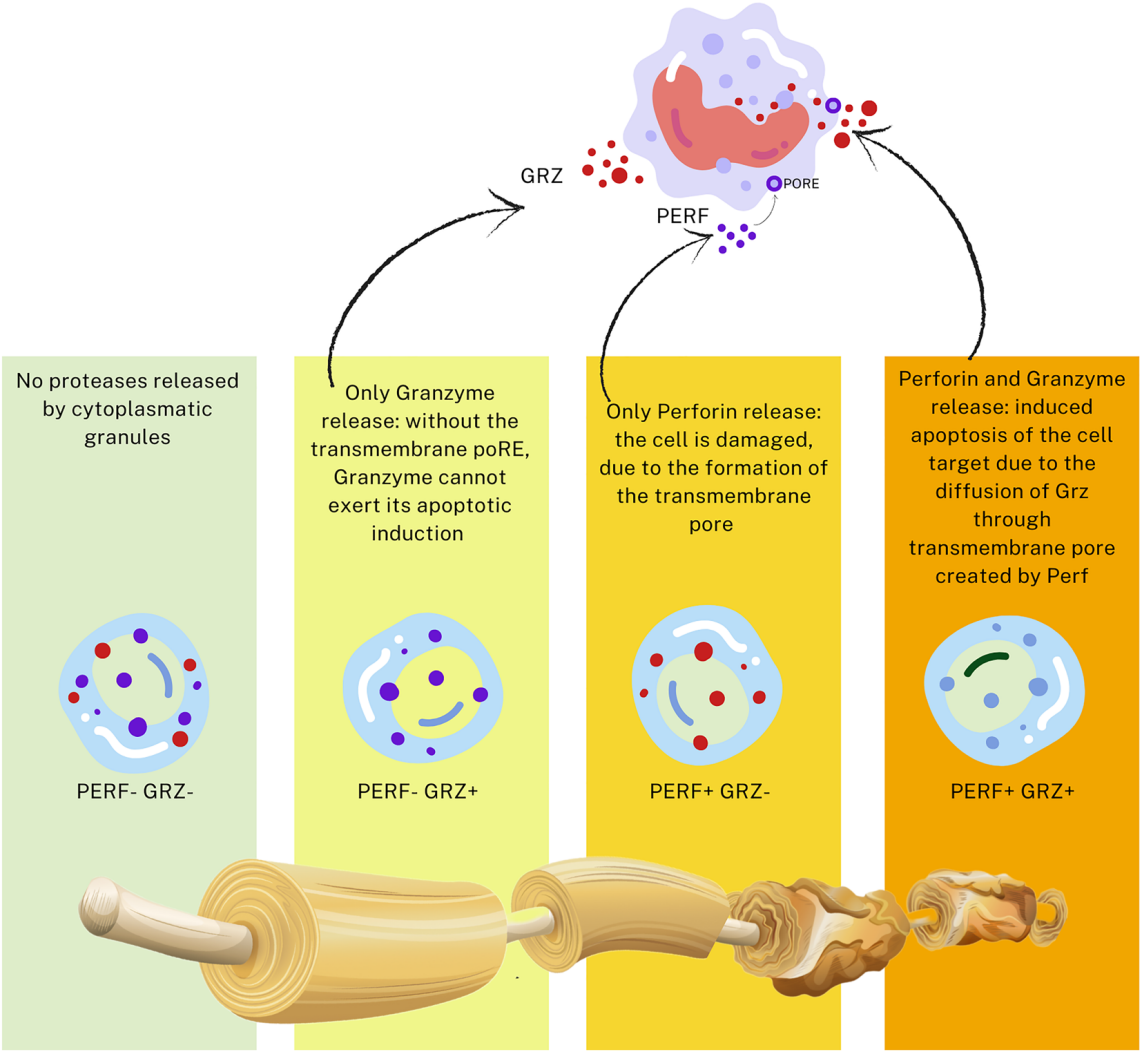
Summary of the Perforin/Granzyme mechanistic hypothesis for CD8^+^ T cells immune-related WM damage in BD. It is surmised that the release of Perforin by CTLs could cause a damage to oligodendrocytes, worsened by Granzyme, while Granzyme alone could induce a minor damage; and while the frequency of CD8^+^ T naïve cells not expressing toxins associates with preserved WM integrity.

CD8^+^ T cells carry out cytotoxic activity, releasing killer proteins^50^. Within these toxins, Perforin and Granzyme are the most important to induce target cell death: the secretory granules migrate along the microtubule-organizing center and release their cytotoxic content in the synaptic cleft formed with the target cell^51^. Perforin is a glycoprotein able to polymerize and form a channel in the membranes of target cells. The opening of a channel disrupts the cell membrane and promotes cell death by affecting the integrity of the target cell^52,53^ and allowing the influx of Granzymes, thus inducing the activation of pro-apoptotic pathways and DNA degradation^54,55^. Animal studies showed that while knockout of Perforin abolished granule-dependent target cell death, disruption of granzyme genes had far smaller consequences^56^. Our results of a general pattern of lower FA and higher RD in association with cells expressing Perforin, and higher FA and lower RD in those not expressing Perforin and Granzyme, suggests that cell damage following CTLs activation may be a mechanism involved in disrupting WM microstructure in BD, with effects distributing along the gradient of toxins expression (Fig. 3).

The activation status of the cells could interact with toxins expression in influencing WM microstructure. CM cells, but not EM and Naïve cells, are associated to negative effects (reduced FA, increased MD and RD) also when only Granzyme is expressed. During an inflammatory response, EM cells show faster effector properties and represent the first line against pathogens, while CM show higher proliferative potential and differentiate into new effectors to maintain the immune response's efficiency^57^. CD8^+^ CM cells secreting cytolytic molecules after restimulation exert a potent effector function^58^ and have a great migratory response, leading to strategic positioning at pathogens entering sites or damage location^59^. CD8^+^ CM cells are also able to self-renewal and re-expansion^60^, and to prompt activation of Naïve CD8^61^. Granzymes can directly influence inflammation^62^ either by directly activating the proinflammatory cytokine IL-1β^63^ or by activating macrophages to secrete cytokines^64^, and can then contribute to immune-neurotoxicity, excitation, and autoimmunity in the brain^65,66^. Sustained production of Granzyme by CM cells could then be possibly sufficient to trigger inflammatory mechanisms, which have been associated with WM damage in BD^31,67^, while Perforin could also be necessary to cause the damage associated with Naïve, EM, and TEMRA cells.

These findings seem, therefore, to support the hypothesis that CD8^+^ T cells can leave the bloodstream to migrate into the brain and induce immune-related WM damage in BD^38^. This mechanism has been described in other conditions associated with WM damage. CD8^+^ T cells are the most prevailing lymphocyte population in inflammatory lesions of patients with multiple sclerosis^68^, and it has been long known that oligodendrocytes are vulnerable to attack by Perforin, which may cause transient or irreversible myelin injury^69^. CTLs play a core role in limbic encephalitis^70^, and animal models confirmed that they can attack oligodendrocytes^71^, particularly in the presence of brain infection^72^, but also cause sclerosis-like lesions by recognizing antigens on oligodendrocytes^73^. It is widely recognized that CD8^+^ T cells contribute to the initiation, progression, and regulation of several human pathogenic autoimmune responses^74^. Recently, Magioncalda and Martino proposed an interesting model suggesting that immune alterations in BD patients may represent the preeminent pathophysiological mechanisms underpinning WM microstructural modifications, especially in the limbic circuit^75^. The immune-mediated WM damage involving the connections within the limbic network would destabilize the neurotransmitter signaling by promoting functional alterations of the neurotransmitter-related nuclei, leading to phasic reconfigurations of intrinsic brain activity. These phasic changes in the activity of brain regions within the limbic network would then manifest as the changes in mood and emotion processing characteristically observed in BD^75^. In agreement with this model, we observed that, irrespective of the illness phase, immune cell populations associated with WM microstructure in several fiber tracts that connect the limbic system with other brain regions, such as the corpus callosum, which connects, amongst others, the anterior cingulate cortex and orbitofrontal cortex playing an essential role in mood regulation^76^; and the corona radiata and the thalamic radiation which are part of the limbic-thalamo-cortical circuitry and also plays an important role in emotion regulation^77^.

*Post-mortem* studies in patients with BD detected apoptotic markers in limbic and prefrontal regions^78,79^, where the density of neurons and glia were decreased^80^. Blood–brain barrier dysfunction has been associated with BD, with leakage predicting more severe illness outcomes^81^. A single, pivotal *post-mortem* study in patients with mood disorders or schizophrenia described diffuse brain cortical and subcortical infiltration by CD8^+^ T cells in 4 out of 9 patients with BD^11^: if this happens during active illness episodes, or is a trait of the illness, remains unknown. Studies in inflammatory conditions with brain damage and CD8^+^ involvement, such as multiple sclerosis, reported lower frequencies of circulating CD8^+^ T cells, paralleling immune activation, autoreactivity, and susceptibility to infections^82^. A tempting hypothesis, formulated in early studies about WM damage in BD but still to be explored in longitudinal studies of patients in euthymic conditions, is that the waxing and waning of illness phases and inflammation, associated with BD, may parallel a pattern of immune-cell mediated WM damage associated with active illness phases^12^. Here cells frequencies are not associated with the number of episodes and illness duration: for this reason, it seems plausible that an inflammatory process is related to the disorder itself, rather than the severity measured as episode number and duration. This seems to be supported by previous findings reporting alterted inflammatory indexes already in adolescents with BD^83^, and not only in adults or patients with years of illness durations.

Finally, the counts of CD8^+^ Naïve cells negative for Granzyme and Perforin, and total CD8^+^CM cells irrespective of toxins expression, had opposite effects on FA: positive for Naïve cells, also associated with a reduction of RD, and negative for CM cells. An expansion of senescence-associated cells (CD8^+^ CD28^−^) in BD^84^ has been reported in the frame of a lifetime pattern of reduction of naïve T cells with expansion of memory and senescent T cells^2–6^. We did not study specific senescence markers, but the finding of a positive effect of Naïve cells on WM integrity, paired with a detrimental effect of CM, suggests that immune-senescence correlates with WM microstructure in BD. Supporting this hypothesis of WM alterations related to lymphocyte cytotoxic activity associated with senescence, Moreno-Valladares, and colleagues found a low-grade infiltration of CD3^+^ and CD8^+^ lymphocytes in WM of post-mortem brain of healthy donors, with increased infiltration in elderly subjects (65–87 years) compared to younger (36–58 years) individuals^85^. Moreover, Magioncalda and colleagues compared the association of characterized CD4 and CD8 cell levels with WM integrity index in healthy controls and BD patients^86^. They found an association between FA and RD measured in the body of the corpus callosum and superior corona radiata only in the manic subgroup of BD patients, where no association was found for HC. Despite our lack of a control group, based on these other findings, we can argue that the baseline differences between HC and BD patients in lymphocytes and CD8 subpopulation levels^87^, significantly associated with measures of WM integrity only in BD patients, thus supporting the idea of a detrimental effect of cytotoxic lymphocytes infiltration in brain of BD patients. Surely, due to the associative nature of MRI studies, the hypothesis of a specific association of cytotoxic CD8 cells and WM measures in BD needs to be tested in future studies, also assessing specific senescence markers.

Strengths of the present study include a focused research question and state-of-the-art imaging methods, but our results must be viewed in light of some limitations. The lack of a healthy control group for DTI limits generalizability to the general population and hampers the possibility of fully understanding immune cell abnormalities and their association with WM microstructure, future studies should address this issue. No patient was drug-naive, and the drug treatments administered during the course of the illness, including antidepressant and antipsychotic drugs, could have influenced MRI measures. Recruitment was in a single center and in a single ethnic group, thus raising the possibility of population stratification. The findings are associative, and a possible causal effect of cytotoxic T-lymphocyte subpopulations must be investigated in experimental settings (e.g., animal studies and or in vitro studies) capable of showing causative relations. These limitations, however, do not bias the main finding of an effect of circulating CD8^+^ cytotoxic cell count on WM integrity in BD, thus providing new insight into possible pathogenetic mechanisms and targets for treatment.

### Supplementary Information

Supplementary Information.

## Data Availability

The datasets used and/or analyzed during the current study are available from the corresponding author on reasonable request.
