# Supplementary material for: Circulating cytotoxic immune cell composition, activation status and toxins expression associate with white matter microstructure in bipolar disorder
Source: Sci Rep. 2023 Dec 14;13:22209. doi: 10.1038/s41598-023-49146-6 (PMC10721611; doi:10.1038/s41598-023-49146-6)
Supplement: Supplementary file 1 — Supplementary Information. [file 41598_2023_49146_MOESM1_ESM.docx]

**SUPPLEMENTARY MATERIAL**

Staining procedure and FACS analysis:

To be analyzed, cryo-preserved PBMCs were removed from liquid nitrogen contained and rapidly thawed in a 37°C water bath (1-2 minutes) to minimize any cell membrane damage. Then, PBMCs were washed by centrifugation and resuspended in RPMI 1640 medium (Biowhittaker) previously supplemented with 10% FBS, 1% Penicillin Streptamycin, and 1% L-Glutamine. PBMCs were stimulated with a solution made of the activators phorbol 12-myristate 13-acetate (PMA) and Ionomyicin from Streptomyces Conglobatus, prepared with an intermediate dilution series. An unstimulated control was performed to control for the efficacy of the PMA/Ionomycin stimulation. After 2 hours of stimulation, cells were resuspended, and BD GolgiPlug protein transport inhibitor-containing Brefeldin A (BD Cytofix/Cytoperm Plus Kit with BD GolgiPlug) previously thaw and diluted were added to retain cytokines within the cells; then, cells were incubated again at 37°C CO2. After the incubation time, the 96-well plate was washed with staining buffer (Stain Buffer – FBS, BD Pharmingen). After the stimulation, we proceeded with the staining protocol. To assess cell viability, cells were marked with the LIVE/DEAD Fixable Aqua Dead Cell Stain (Invitrogen, Thermo Fisher), previously reconstituted with DMSO and diluted with PBS 1X. For both the intracellular and surface staining, a specific mix of primary fluorochrome-conjugated antibodies was prepared and subsequently added. The surface staining mix was prepared with Stain Buffer – FBS, BD Pharmingen, and the BD Perm/Wash Buffer was used to prepare the intracellular antibodies mix. The mixtures were stored covered from light exposure at 4° until their use. Then, surface antibodies mix was added, and the 96-well plate was incubated at 4°C protected from light exposure. Cells were then washed with staining buffer and we proceeded with the intracellular staining. Cells were fixed and permeabilized with the Fixation/Permeabilization solution of the BD Cytofix/Cytoperm Plus Fixation/Permeabilization Kit with GolgiPlug was used (BD Biosciences). Then, cells were washed two times with the diluted Perm/Wash Buffer to completely remove all the buffers and solutions from cells. Once cells were fixed and permeabilized, the previously prepared antibodies mix was added, and stored at 4°C; then, the diluted Perm/Wash Buffer (BD Biosciences) was added. At this point, all the surface and intracellular/intranuclear staining were completed: staining buffer (Stain Buffer – FBS, BD Pharmingen) was added to each well to gently resuspend cells and then transferred into 5mL round-bottom polypropylene tubes (Falcon). Then, all the tubes were transferred, covered from light exposure, to the FACS facility to be analyzed with the BD FACSymphony A5 Cell Analyzer (BD Biosciences). This novel high-parameter cell analyzer supports up to 50 high-performance photomultiplier tubes (PMTs), improving detection sensitivity. Acquired data were analyzed through the FlowJo v 10.7.1 software (Tree Star Inc, Ashland, Oregon, USA). Each experiment was preceded by 8-peak Rainbow calibration particles (BioLegend) acquisition to evaluate instrument performance and to standardize instrument settings for all the acquisition performed, regardless of different acquisition days. Moreover, Isotype controls were used with low expression populations, such as cells releasing Perforin and Granzym, to ensure that the observed staining is due to the specific antibody binding rather than an artifact.

**Supplementary Figure 1**. The gating strategy used for characterized cytotoxic T-cells, NK, and Tγδ subpopulations.


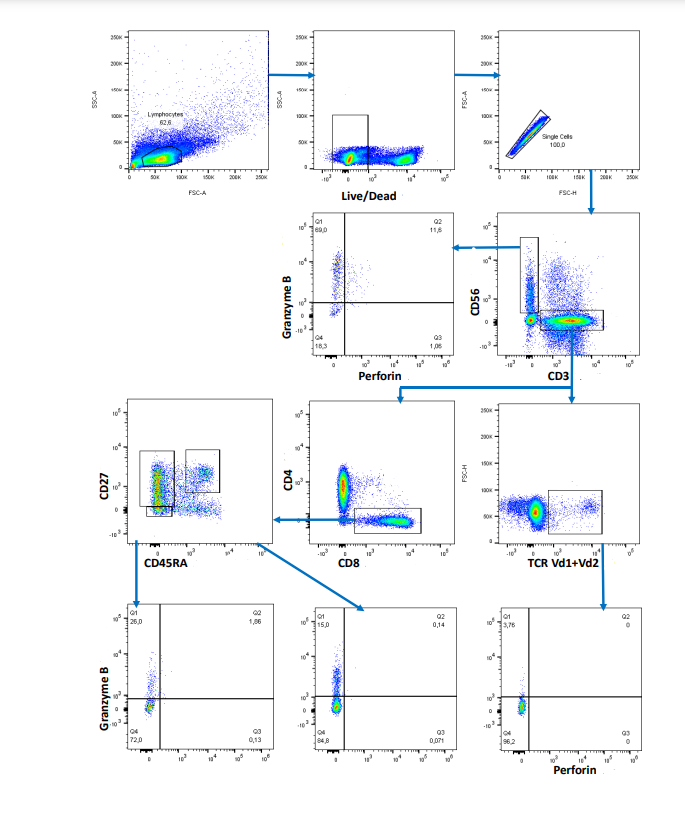


**Table 1.** Direction of the significant effects of frequencies of circulating cytotoxic T lymphocytes, divided according to lineage and activation status of the cells, on measures of WM microstructure. ↑=increased; ↓=decreased; -=no effect.

| Activation status | FA | MD | RD |
| --- | --- | --- | --- |
| Naïve CTLs |  |  |  |
| CD3^+^ CD8^+^ Naïve Perf^-^Grz^-^ | ↑ | - | ↓ |
| CD3^+^CD8^+^Naïve Perf^+^Grz^-^ | ↓ | ↑ | ↑ |
| CD3^+^CD8^+^Naïve Perf^+^Grz^+^ | ↓ | - | ↑ |
| Effector Memory CTLs |  |  |  |
| CD3^+^CD8^+^EM Perf^-^Grz^+^ | ↑ | - | ↓ |
| CD3^+^CD8^+^EM Perf^+^Grz^-^ | ↓ | - | ↑ |
| CD3^+^CD8^+^EM Perf^+^Grz^+^ | ↓ | - | - |
| Central Memory CTLs |  |  |  |
| CD3^+^CD8^+^CM (all) | ↓ | - | - |
| CD3^+^CD8^+^CM Perf^-^Grz^-^ | - | ↓ | ↓ |
| CD3^+^CD8^+^CM Perf^+^Grz- | ↓ | - | ↑ |
| CD3^+^CD8^+^CM Perf^-^Grz^+^ | ↓ | ↑ | ↑ |
| CD3^+^ CD8^+^CM Perf^+^Grz^+^ | ↓ | - | - |
| TEMRA |  |  |  |
| CD3^+^CD8^+^TEMRA Perf^-^Grz^+^ | ↑ | - | - |
| CD3^+^CD8^+^TEMRA Perf^+^Grz^-^ | ↓ | - | ↑ |
